# Multicentre study on the accuracy of lung ultrasound in the diagnosis and monitoring of respiratory sequelae in the medium and long term in patients with COVID-19

**DOI:** 10.3389/fmed.2023.1199666

**Published:** 2023-05-25

**Authors:** Cristina Ramos Hernández, Amara Tilve Gomez, Ana Sánchez Fernández, Rosa Cordovilla, Ana Núñez Ares, Paola Ordoñez Gómez, Aurelio Wangüemert Pérez, Olalla Castro Anón, Jorge González Ramírez, Mar Valdivia Salas, Javier Pérez Pallares, Diego Ferrer Pargada, Fernando Vargas Ursúa, Irene Lojo Rodriguez, Almudena González Montaos, Maribel Botana Rial, Alberto Fernández Villar

**Affiliations:** ^1^Álvaro Cunqueiro Hospital in Vigo, Pneumology Service, NeumoVigo I + i, Southern Galicia Biomedical Research Institute (IISGS), Vigo, Spain; ^2^Álvaro Cunqueiro Hospital in Vigo, Radiology Service, Vigo, Spain; ^3^Salamanca University Clinical Hospital, Pneumology Service, Salamanca, Spain; ^4^Albacete University Hospital Complex, Pneumology Service, Albacete, Spain; ^5^Valencia University Clinical Hospital, Pneumology Service, Valencia, Spain; ^6^San Juan de Dios Hospital in Tenerife, Pneumology Service, Tenerife, Spain; ^7^Lucus Augusti Hospital, Pneumology Service, Lugo, Spain; ^8^Lucus Augusti Hospital, Radiology Service, Lugo, Spain; ^9^Santa Lucía de Cartagena General University Hospital, Pneumology Service, Cartagena, Spain; ^10^Marqués de Valdecilla Hospital, Servicio de Neumología, Pneumology Service, Valencia, Spain

**Keywords:** LUS, elastography, ultrasound, COVID-19, SWE

## Abstract

**Introduction:**

Lung ultrasound (LUS) has proven to be a more sensitive tool than radiography (X-ray) to detect alveolar-interstitial involvement in COVID-19 pneumonia. However, its usefulness in the detection of possible pulmonary alterations after overcoming the acute phase of COVID-19 is unknown. In this study we proposed studying the utility of LUS in the medium- and long-term follow-up of a cohort of patients hospitalized with COVID-19 pneumonia.

**Materials and methods:**

This was a prospective, multicentre study that included patients, aged over 18 years, at 3 ± 1 and 12 ± 1 months after discharge after treatment for COVID-19 pneumonia. Demographic variables, the disease severity, and analytical, radiographic, and functional clinical details were collected. LUS was performed at each visit and 14 areas were evaluated and classified with a scoring system whose global sum was referred to as the “lung score.” Two-dimensional shear wave elastography (2D-SWE) was performed in 2 anterior areas and in 2 posterior areas in a subgroup of patients. The results were compared with high-resolution computed tomography (CT) images reported by an expert radiologist.

**Results:**

A total of 233 patients were included, of whom 76 (32.6%) required Intensive Care Unit (ICU) admission; 58 (24.9%) of them were intubated and non-invasive respiratory support was also necessary in 58 cases (24.9%). Compared with the results from CT images, when performed in the medium term, LUS showed a sensitivity (S) of 89.7%, specificity (E) 50%, and an area under the curve (AUC) of 78.8%, while the diagnostic usefulness of X-ray showed an S of 78% and E of 47%. Most of the patients improved in the long-term evaluation, with LUS showing an efficacy with an S of 76% and E of 74%, while the X-ray presented an S of 71% and E of 50%. 2D-SWE data were available in 108 (61.7%) patients, in whom we found a non-significant tendency toward the presentation of a higher shear wave velocity among those who developed interstitial alterations, with a median kPa of 22.76 ± 15.49) versus 19.45 ± 11.39; *p* = 0.1).

**Conclusion:**

Lung ultrasound could be implemented as a first-line procedure in the evaluation of interstitial lung sequelae after COVID-19 pneumonia.

## Introduction

The possibility of developing respiratory sequelae in patients who survive pneumonia caused by severe acute respiratory syndrome coronavirus 2 (SARS-CoV-2) has been the subject of study since the start of the COVID-19 pandemic (1, 2). In previous epidemics caused by viruses such as SARS-CoV or Middle East respiratory syndrome (MERS), a variable number of patients developed residual interstitial lung disease (3, 4). Thus, it is crucial to detect possible early markers that can identify patients presenting an unfavorable evolution in an attempt to start early treatments and thereby avoid possible sequelae.

To date, academic publications indicate that respiratory sequelae are present in up to 20–80% of patients after having overcome SARS-CoV-2 pneumonia. This wide variability lies in the different study cohorts considered, disease severity, and treatments administered in the acute phase of the illness (5). At the radiological level, the most frequently reported sequelae are the presence of “ground glass” areas and reticular lesions or parenchymal bands (2, 6). Furthermore, data suggestive of pneumonitis and organizing pneumonia have been described at the histological level (7). Although the possible implications and severity of these sequelae are unknown at this time, these patients must be closely monitored.

Several protocols endorsed by different scientific societies are now available that propose chest radiography (X-ray) as the first imaging technique of choice for monitoring these patients. Thus, computed tomography (CT) is usually reserved for cases presenting persistent changes in the X-ray images after 3 months with the suspicion of thromboembolic disease or persistent respiratory symptoms with normal X-ray imaging (8, 9). However, despite these protocols, until now CT has been the most widely used imaging technique for follow-up of these patients because it unquestionably detects small residual anomalies such as a those with a ground glass appearance, with better accuracy than X-ray imaging (10).

Notwithstanding, CT is an expensive technique which emits ionizing radiation and its use in these cases implies a care overload for radiology services. The ideal tool for this type of monitoring would be inexpensive, accessible at the bedside, and would not emit ionizing radiation (such as ultrasound). However, none of the protocols available to date includes lung ultrasound (LUS) as a follow-up in patients with respiratory sequalae from SARS-CoV-2.

During the COVID-19 pandemic, LUS proved to be a useful tool not only for establishing an early diagnosis of COVID-19 pneumonia, but also for predicting the evolution of these patients and detecting possible complications of the disease (9, 11, 12). Given the characteristics of this technique, it could also be an ideal tool as the first diagnostic test used in the medium and long-term follow-up of these patients. Given all the above, the objective of this study was to describe the diagnostic accuracy of LUS, in the medium and long term, for the early detection of alterations in the lung interstitium compared to chest CT imaging, which is currently considered the gold standard.

## Materials and methods

### Objectives

The main objective of this study was to analyze the accuracy of the ultrasound findings to detect alterations and/or respiratory sequelae in the medium and long term after COVID-19 pneumonia. The secondary objectives included defining ultrasound abnormalities in the follow-up of COVID-19 pneumonia, as well as evaluating whether recently developed techniques such as two-dimensional shear wave elastography (2D-SWE) could help the early detection of interstitial sequelae.

### Study design and participants

This was an observational and prospective multicentre study in which the following national hospitals participated: Hospital Álvaro Cunqueiro de Vigo, Hospital Clínico Universitario de Salamanca, Complejo Hospitalario Universitario de Albacete, Hospital Clínico Universitario de Valencia, Hospital San Juan de Dios de Tenerife, Hospital Universitario Lucus Augusti, Santa Lucía de Cartagena General University Hospital, and the Marqués de Valdecilla Hospital.

Patients from a specific post-COVID-19 follow-up consultation, aged over 18 years, who had been discharged after a confirmed SARS-CoV-2 infection with pneumonia from April 2020 to March 2021 were included. Patients who, despite having presented a SARS-CoV-2 infection, had not developed pneumonia during the admission and those who refused to participate in the study were excluded. All the participants underwent a general clinical evaluation, radiology studies, and pulmonary function tests at both 3 ± 1 and 12 ± 1 months after hospital discharge.

### Information gathering

Variables related to the admission such as the days of hospitalization and need for non-invasive support or admission to critical care units were collected. In the two visits carried out in person in the medium and long term, the following variables were recorded: clinical variables regarding the presence of cough, expectoration, chest pain, degree of dyspnea determined according to the modified Medical Research Council (mMRC) scale (13), presence of orthopnea, and persistent fever, headache, or muscle weakness. The Nottingham scale was used to assess health-related quality of life (14).

### Functional testing

At the medium and long-term visits, forced spirometry was performed following the joint recommendations of the American Thoracic Society and the European Respiratory Society (ATS/ERS) (15) and using the Global Lung Function Initiative (GLI) equations (16). Absolute and percentage values of forced vital capacity (FVC), expired volume in the first second (FEV1), and the ratio of both these metrics (FEV1/FVC) were recorded. A single breath diffusing capacity of the lungs for carbon monoxide (DLCO) test was also performed following the ERS/ATS recommendations (17), recording its absolute and percentage value. Finally, a 6-min walk test was performed following the ATS recommendations (18) and the distance traveled and initial and final oxygen saturations, dyspnea measured according to the initial and final Borg scale result, and number of stops during the test were recorded.

### Radiological tests

At the two visits, all the patients underwent a posteroanterior and lateral chest X-ray, reported upon by an expert radiologist. The chest X-ray images were classified as normal, persistent lesions after COVID-19 pneumonia, or in the event that an existing alteration had been detected before the SARS-CoV2 infection, as unchanged. All the patients presenting mMRC grade ≥ 2 dyspnea, radiographical, ultrasound, or spirometry abnormalities, and/or DLCO < lower limit of normality (LLN) underwent a high-resolution chest CT in the subsequent 2 weeks. Following the recommendations of the Fleischner Society guidelines, the CT images were analyzed by expert radiologists who were unaware of the clinical functional status of the patients (19). CT was considered the gold standard diagnostic technique. The presence of ground glass opacities, reticular lesions, bronchiectasis, and honeycomb patterns were recorded. A quantification of the extension of the lesions visualized in the CT in the five pulmonary lobes was also performed in a subgroup of patients (20, 21).

### Lung ultrasound

**FIGURE 1:**
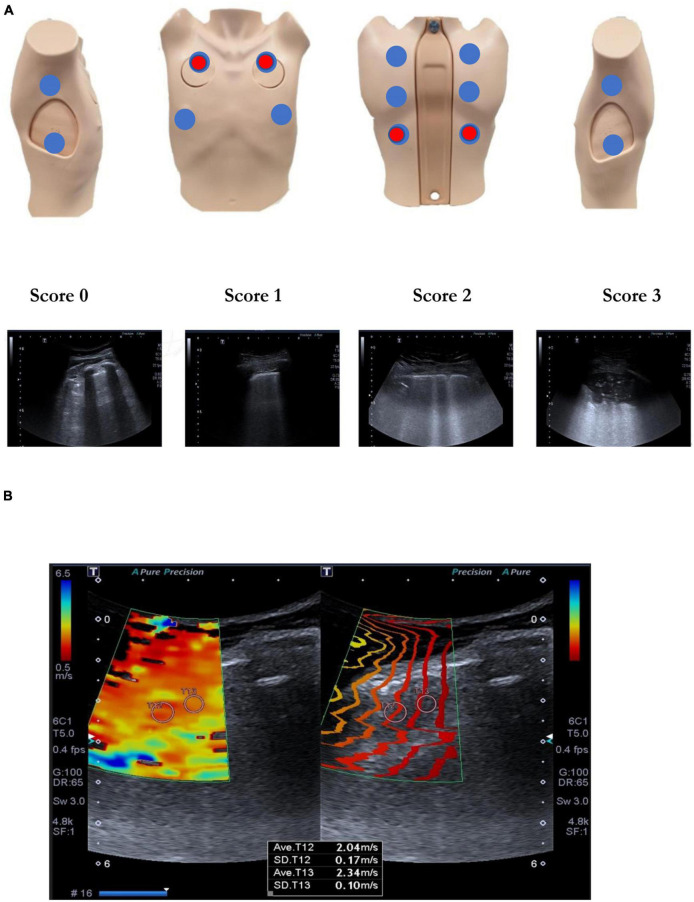
**(A)** Lung ultrasound. Exploration protocol of 14 areas and scoring for each area. In red the elastographic assessment points. **(B)** Lung 2D-SWE elastography.

Lung ultrasound examinations were performed on all patients both at the first visit (3 ± 1 months) and at the second visit (12 ± 1 months). A 2–5 MHz convex probe was used for this exploration and the protocol consisted of a complete assessment of all intercostal spaces, which were divided into 14 areas (Figure 1A), following the recommendations of the International Consensus Document on Lung Ultrasound for COVID-19 patients (22). Each of the explored areas was scored based on the alterations detected, with 0 assigned if the parenchyma was adequately aerated with the presence of A lines; (1) where at least three vertical hyperechogenic artifacts (starting from the pleural lines and without extinguishing at the end of the screen, known as B lines) were present; (2) where the B lines tended toward coalescence; and (3) when there was an area of white lung or consolidation of the lung parenchyma. The sum of the scores of each of the 14 areas was recorded as the “lung score.” Altered LUS results were defined as all those with a lung score ≥ 1 while normal LUS findings were those with a lung-score of 0 without the presence of artifacts in the pleural line.

In a subgroup of patients, the elastic properties of the lung tissue were also analyzed using 2D-SWE during apnea after having performed a maximum inspiration. In this case, 4 areas were explored: 2 anterior areas and 2 posterior areas (Figure 1B). At least 3 valid measurements were made of each of the explored spaces and a qualitative assessment was recorded by Itho elastogram, a semi-quantitative assessment technique (by measuring the A/B hardness ratio and comparing the hardness of the lung parenchyma with respect to that of the chest wall) as well as quantitative assessments through the mean kPa.

### Statistical analysis

Qualitative variables were reported as the frequency and percentage, while quantitative variables were reported as the mean (± standard deviation, *SD*) in the case of a normal distribution or as the median (± interquartile range, IQR) for non-normally distributed data. We used the mean of the difference and its 95% confidence interval (CI) to express the differences between the studied parameters. The quantitative variables were compared using Student *t*-tests for paired samples, with *p*-values equal to or less than 0.05 being considered statistically significant. The validity and safety of LUS for the detection of sequelae using the standard equations as well as the sensitivity (S), specificity (E), positive predictive value (PPV), and negative predictive value (NPV). ROC curves were plotted to analyze the area under the curves (AUCs). All the statistical analyses were performed with IBM SPSS Statistics software (version 21, IBM Corp., Armonk, NY, USA). The study was approved by the Galician Clinical Research Ethics Committee (registration number 2020/245) in April 2020.

## Results

**FIGURE 2:**
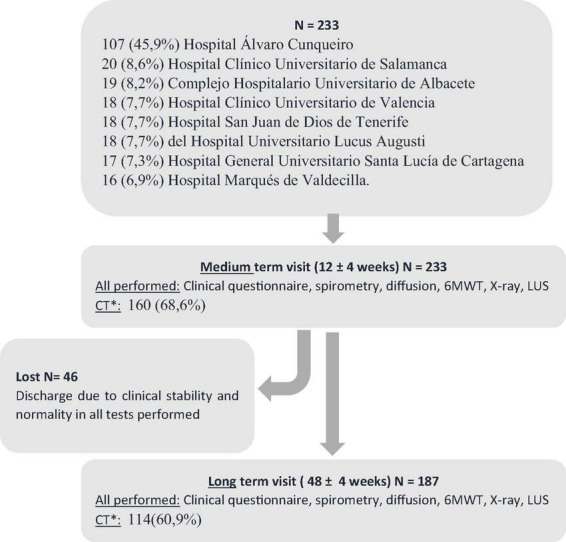
Flow chart. *CT performed in all those with: mMRC grade ≥ 2 dyspnea or abnormalities in X-ray, lung ultrasound, spirometry and/or DLCO < LLN. The high-resolution chest CT was performed in the following 2 weeks. Computerized axial tomography (CT), 6-min walk test (6MWT), radiography (X-ray), lung ultrasound (LUS).

A total of 233 patients were included of whom, 107 (45.9%) were from the Álvaro Cunqueiro de Vigo Hospital (Figure 2). The mean age of the patients was 62.4 years (IQR 54–71), with 150 (64.4%) being male. Of the total sample, 76 (32.6%) patients required admission to the ICU, of which 58 (24.9%) were intubated, and extracorporeal membrane oxygenation (ECMO) was used in 3 cases (1.3%). Non-invasive respiratory support was necessary in 58 cases (24.9%). The severity of the pneumonia according to the FINE prognostic scale (23) at admission it was 56.2 (*SD* = 37.5) points and that of CURB-65 (24) was of 1.01 (of 0.8). The mean stay during hospital admission was 17.6 (IQR = 7–22) days. The clinical and functional characteristics collected in the mid- and long-term visits are shown in Table 1.

**TABLE 1:** Sample characteristics at 3 and 12 months of assessment.

	12 ± 4 weeks after discharge (SD)	48 ± 4 weeks after discharge (SD)	*p*
**Clinical features**			
mMRC dyspnea	1.88 (0, 80)	1.63 (0, 82)	0.001
Cough	2.84 (1, 40)	1.72 (1, 24)	0.001
Expectoration	1.18 (0, 38)	1.07 (0, 25)	0.001
Chest pain	1.15 (0, 37)	1.10 (0, 48)	0.90
Muscular weakness	2.76 (0, 36)	2.03 (0, 29)	0.001
Myalgias	2.31 (1, 36)	1.70 (1, 39)	0.001
Headache	1.65 (1, 40)	1.28 (1, 25)	0.001
Nausea	1.05 (0, 25)	1.01 (0, 09)	0.10
Diarrhea	1.01 (0, 09)	1.01 (0, 09)	1
Lower limbs edema	1.10 (0, 30)	1.05 (0, 21)	0.03
Orthopnea	1.06 (0, 24)	1.01 (0, 09)	0.01
**Nottingham scale** (14)			
Energy	0.60 (0, 89)	0.48 (0, 88)	0.06
Pain	1.51 (2, 17)	1.21 (1, 95)	0.01
Mobility	1.55 (1, 82)	1.16 (1, 71)	0.001
Emotional reactions	1.11 (1, 74)	1.13 (1, 84)	0.88
Sleep	1.20 (1, 57)	1.15 (1, 48)	0.58
Social isolation	0.31 (0, 70)	0.28 (1, 15)	0.69
**Laboratory tests**			
Leukocytes	1817.04 (3132, 05)	1684.25 (2805, 71)	0.06
Lymphocytes	456.59 (844, 29)	591.77 (581, 15)	0.11
Platelets (×10^3^)	121.46 (297, 85)	71.87 (107, 10)	0.01
PCR	2.87 (9, 4)	3.21 (7, 5)	0.66
D-dimer	475.65 (650, 06)	415.68 (491, 44)	0.68
LDH	226.63 (153, 33)	204.83 (97, 89)	0.06
Ferritin	259.96 (511, 75)	149.60 (152, 39)	0.01
**Functional testing**			
FEV1%	94.51 (19, 01)	97.76 (18, 52)	0.001
FVC%	93.96 (18, 46)	98.29 (15, 92)	0.001
FEV1/FVC	92.82 (103, 32)	77.88 (7, 86)	0.36
DLco	77.37 (18, 74)	85.98 (19, 91)	0.001
TM6M distance (m)	436.99 (118, 89)	484.33 (105, 31)	0.001
SpO2 delection in 6MWT	2.78 (2, 94)	2.27 (2, 56)	0.01
**Imaging Tests**			
Altered CT	57.1%	39.9%	0.04
Altered X-ray	50.6%	11%	0.02
Lung-Score	5.80 (5, 23)	2.71 (3, 88)	0.001
Elastography			
Ratios A/B	4.97 (24, 23)	1.31 (0, 34)	0.27
Kpa (media)	21.91 (13, 73)	20.62 (11, 49)	0.52
Itho	1.07 (1, 17)	0.84 (0, 94)	0.12

**FIGURE 3:**
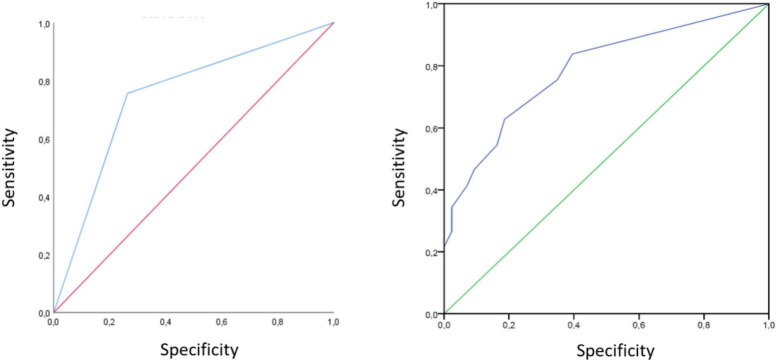
Lung ultrasound ROC curves in the medium and long term visits. Area under ROC curve to determine the LUS ability to discriminate patients with interstitial lung sequelae after COVID-19 pneumonia, compared with chest CT in the mid **(left)** and long term **(right)** visit follow up.

The first visit was measured 2.93 (*SD* = 1.32) months after discharge. LUS was performed on this visit in all the participants (233) and the results were altered in 168 cases (72.1%). The mean lung score was 5.3 (*SD* = 5.1) and the most affected areas were detected in the subsequent exploration of the right lower lobe 0.75 (*SD* = 0.82) and of the left lower lobe 0.56 (*SD* = 0.70). A CT was performed in 160 (68.6%) of these patients and was normal in 27 (11.6%); in the remaining patients residual lesions persisted after COVID-19 pneumonia. LUS, performed in the medium term and compared with CT results, showed a sensitivity of 89.7%, specificity of 50%, and PPV of 90%. The Kappa index, a statistical measure of inter-rater reliability, was 0.73 and AUC was 78.8% (Figure 3). On the other hand, the diagnostic efficacy of X-ray versus CT showed a sensitivity of 78%, specificity of 47%, and PPV of 89%. At this visit, a sub-analysis was performed on 84 patients to assess the type of alterations seen in the CT and its extension studies (Table 2). This analysis indicated that the lung score allowed the detection of different types of radiological alterations, regardless of the degree of involvement.

**TABLE 2:** Sub-analysis of the CT abnormalities.

	*N*	Lung-score (SD)	*P*
Ground glass			*P* = 0,000
< 25%	58	3.93 (3, 66)	
≥ 25%	26	9.54 (9, 54)	
Ground glass			*P* = 0,000
< 5%	37	3.46 (3, 71)	
≥ 5%	47	7.40 (5, 71)	
Reticulation			*P* = 0,000
Yes	60	4.15 (4, 05)	
No	24	9.46 (6, 15)	
Crazy-paving			*P* = 0,917
No	82	5.65 (5, 25)	
Yes	2	6.50 (9, 19)	
Fibrosis			*P* = 0,001
No	75	4.8 (4, 21)	
Yes	9	14.1 (5,86)	

The second visit was conducted 11.2 (*SD* = 2.65) months after discharge. LUS was performed on all the participants and showed a significant improvement in the lung score compared to the first visit (5.8 ± *SD* 5.2 vs. 2.1 ± *SD* 3.8; *p* = 0.001). In this group of patients, a new CT was performed in 114 cases (60.9%), with the results being normal in 19 cases (11%), while residual lesions attributable to COVID-19 persisted in the remaining cases. The sensitivity of LUS at this visit was 76%, specificity was 74%, PPV was 93%, and the AUC was 0.74 (Figure 3). While the diagnostic yield of the X-ray showed a sensitivity of 71%, specificity of 50%, and PPV of 88%.

Two-dimensional shear wave elastography data were available for 108 (61.7%) patients, in whom we found a non-significant tendency toward the presentation of a higher shear wave velocity in those who had developed interstitial alterations, with a median in kPa of 22.76 ± 15.49 versus 19.45 ± 11.39 (*p* = 0.1), A/B ratios of 4.53 ± 18.94 versus 2.47 ± 3.60 (*p* = 0.42), and an Itho elastogram result of 1.8 ± 1.27 versus 1.64 ± 1.20 (*p* = 0.34).

## Discussion

This study is the first published to date to evaluate LUS in the medium and long term for the detection of sequelae associated with COVID-19 pneumonia. Compared to CT, the presence of pathological B lines detected by LUS was adequately able to discriminate these persistent abnormalities, which supports the use of LUS as a first-line procedure to rule out pulmonary sequelae after COVID-19 pneumonia.

It is striking that LUS has been considered by different scientific societies as one of the key imaging tests in the acute phase of the disease, both to establish the diagnosis (11) and its prognosis (25). However, the protocols (8, 26) published to date for the follow-up of possible sequelae recommend X-ray as the first imaging test to be used. Only the ERS consensus (10) mentions the possibility of using LUS in patients who presented a more serious clinical course or in those who present new or progressive respiratory symptoms in medium–long term follow-up after COVID-19 pneumonia. However, they report that in these cases it would be useful to perform a low-dose CT as the first diagnostic choice and when not possible, to then use LUS or X-ray interchangeably.

Our results agree with those published by other series in which patients were evaluated at 3 (27), 2–5 (28), and 6 (29) months after hospitalization. Only one published article (30) evaluated sequelae with ultrasound in the medium (1–5 months) and long (5–13 months) term, but it was a single-center study that had included only 16 patients and did not compare the results with CT images as the gold standard. Hence, these researchers could only conclude that the lung score had reduced by 46% from the first visit to the second one—the same reduction we observed in our sample. Thus, to the best of our knowledge, this current work is the first study published to date that evaluates the reliability of LUS and compares it with the gold standard of CT in the medium and long term and in a multicentre manner, thereby increasing its validity.

Despite the improvement in the lung score during follow-up, 93 (39.9%) patients continued to present interstitial changes on the CT imagining 1 year after hospital discharge, despite the fact that only 32% of the sample had presented severe pneumonia in the initial acute phase. These data suggest that the interstitial abnormalities associated with COVID-19 may take several months to resolve and that their resolution does not depend exclusively on the severity of the symptoms. Therefore, it is essential to develop a tool that dynamically allows close monitoring of all patients at the bedside for post-COVID-19 consultations. Given the progressive improvement in radiological techniques, the detection of alterations is increasingly subtle and therefore both X-ray and LUS lose sensitivity over time compared to CT. Nonetheless, in this work, LUS continued to be superior to X-ray at all time points during follow-up.

In addition, elastography is a non-invasive tool to measure the stiffness of superficial lung tissue. Changes in shear wave velocities could imply an early change in the hardness of the underlying tissue and seems to be a promising tool for the detection of interstitial diseases (31–33). In this regard, in our study we did not obtain significant results for the detection of sequelae after COVID-19. However, we believe that in order conduct comparative studies and promote the development of this tool, we must first standardize the technique, describe the number of measurements needed in each area, define the acceptable level of variability to be able to describe a measurement as reliable, and decide whether the test should be performed for the total lung capacity or the residual volume.

Lung ultrasound is already a highly sensitive tool, but to improve its specificity it would be interesting to integrate it into predictive clinical models. In this sense, the RELIC scale (34) includes clinical, analytical, and ultrasound parameters to predict sequelae after COVID-19 pneumonia. Among the items with a higher score, the presence of ultrasound abnormalities 2–4 months after discharge or the need for mechanical ventilation for more than 14 days stands out in the acute phase for patients with medium to long term COVID-19 sequalae and showed a specificity of 91% to detect possible interstitial alterations after COVID-19.

Of note, our study had some limitations such as the non-consecutive inclusion of patients, which may imply that there was a selection bias in this work. This was because of the high level of care overload the COVID-19 pandemic caused, which we tried to compensate for by using a multicentre methodology. Another limitation was the fact that CT results were not available for all the patients included in this study, which could have led to an increase in false negatives for LUS. However, given that CT scans were performed in all patients with dyspnea, or functional, radiological, or ultrasound abnormalities, we believe that the detection of lesions in CT scans that do not impact clinical, radiological, or functional abnormalities was of little significance in the management of these patients.

Nonetheless, this work had numerous strengths such as its multicentre design, which made it possible to achieve an adequate sample size and a representative sample of the population, including patients who presented pneumonia at different stages of severity during the acute phase while in follow-up. In addition, assessment of the diagnostic usefulness of this tool at different times of the evolution of the disease gave it greater diagnostic power and confirmed other results published to date that demonstrate its important translational power in clinical practice.

## Conclusion

Lung ultrasound could be implemented as a first-line procedure in the evaluation of interstitial lung sequelae after COVID-19 pneumonia in both mid- and long-term evaluations. The identification of pathological B lines showed a high sensitivity for the detection of these anomalies and so a normal lung-score could rule out the presence of interstitial disease, avoiding the need for additional diagnostic tests such as CT scans.

## Data availability statement

The raw data supporting the conclusions of this article will be made available by the authors, without undue reservation.

## Ethics statement

The studies involving human participants were reviewed and approved by the Comité de Ética de la Investigación con Medicamentos de Galicia (CEIm-G). The patients/participants provided their written informed consent to participate in this study.

## Author contributions

All authors listed have made a substantial, direct, and intellectual contribution to the work, and approved it for publication.
